# Balancing the benefits and risks of social media on adolescent mental health in a post-pandemic world

**DOI:** 10.1186/s13034-025-00951-z

**Published:** 2025-08-13

**Authors:** Augustus Osborne

**Affiliations:** Institute for Development, Western Area, Freetown, Sierra Leone

## Abstract

The COVID-19 pandemic intensified adolescents reliance on social media for connection, education, and entertainment, presenting both opportunities and risks for mental health. This viewpoint explores the dual nature of social media as a lifeline offering peer support and access to resources, especially for marginalized teens and a stressor, linked to anxiety, depression, and cyberbullying. Drawing on global evidence, including WHO and UNICEF data, it shows disparities in impact across socioeconomic, cultural, and gender contexts, with low-resource settings facing unique challenges like digital poverty amidst persistent post-pandemic effects. A multi-stakeholder framework is proposed to balance these dynamics, emphasizing parental and educator empowerment through digital literacy, tech industry accountability via adolescent-specific safeguards, clinical integration of social media screening in healthcare, and robust policy regulation for online safety. The urgency of action is underscored, with specific calls to governments, tech companies, clinicians, and researchers to collaborate on protecting adolescent well-being. This viewpoint argues that transforming social media into a safe space for mental health is a moral imperative, essential to prevent a generational crisis and ensure equity in the digital age.

## Introduction

Imagine a 15-year-old girl in a rural village in Sierra Leone, scrolling through Instagram late at night during the COVID-19 lockdown, her only window to the outside world while schools remained shuttered. The platform offers her a sense of connection, a space to share her fears about the future with peers across the globe. Yet, the same app bombards her with images of unattainable beauty standards, fuelling anxiety that keeps her awake long after her screen dims. Contrast this with a teenage boy in an urban centre who, through a TikTok hashtag, discovers a mental health support group that helps him navigate depression after losing a family member to the virus. Social media, for adolescents, is a paradox a lifeline and a stressor, a tool for empowerment and a trigger for distress.

The COVID-19 pandemic accelerated adolescents reliance on digital platforms for education, socialization, and entertainment [1]. A 2021 Pew Research Centre report found that 80% of teens in high-income countries used social media daily during lockdowns, a trend mirrored, albeit unevenly, in low- and middle-income settings where shared devices or limited internet access still facilitated notable engagement [1]. Adolescents, aged 10–19 years, are uniquely vulnerable during this developmental stage, as their brains undergo rapid changes in areas governing emotion regulation and identity formation, making them particularly susceptible to external influences like social media [2]. Recent global estimates indicate a substantial increase in depressive and anxiety disorders among adolescents during the COVID-19 pandemic, with over 53 million additional cases of major depressive disorder and 76 million additional cases of anxiety disorders reported worldwide in 2020 alone [3]. The youth mental health crisis is now recognized as a global public health emergency, with evidence-based strategies needed to address both prevention and intervention [4].

This viewpoint confronts a pressing public health challenge: the mental health crisis among adolescents, exacerbated by social media’s dual nature. On one hand, platforms offer unprecedented access to resources and communities; on the other, they contribute to anxiety, depression, and loneliness through cyberbullying, comparison culture, and sleep disruption [5]. These impacts are not uniform socioeconomic, cultural, and gender disparities shape how risks and benefits manifest, often deepening inequities in well-being. Post-pandemic, as digital habits persist, we cannot ignore this dilemma. This article critically assesses the balance between social media’s benefits and risks for adolescent mental health, proposing a multi-stakeholder framework to mitigate harm while harnessing potential. The findings and recommendations align with recent commentary published in Child and Adolescent Psychiatry and Mental Health, which also highlights the dual impact of digital platforms on youth well-being and underscores the need for multi-sectoral responses [6].

In my work at the Institute for Development in Freetown, Sierra Leone, I have witnessed firsthand how adolescents, even in resource-constrained environments, increasingly turn to social media as a coping mechanism amidst challenges like limited access to education and mental health services post-COVID-19. For instance, in a recent community engagement majority of the teens reported using social media as their primary source of information and peer connection, yet many also said exposures to harmful content as a source of stress. This local reality mirrors global trends but underscores unique barriers in low-income settings that demand tailored solutions.

### Benefits for adolescent mental health

Social media has emerged as a powerful tool for supporting adolescent mental health, particularly for those with limited access to traditional resources. Platforms like Instagram, TikTok, and YouTube provide spaces where teens can access information, share experiences, and connect with peers facing similar struggles [1]. For groups such as LGBTQ + youth, those in rural areas, or adolescents in low-income settings social media offers a rare avenue for community building. A 2020 study in the USA found that 70% of LGBTQ + teens reported finding supportive networks online, reducing feelings of isolation often exacerbated by offline stigma [7]. Similarly, during the pandemic, virtual peer support groups on platforms like Discord became lifelines for teens unable to access in-person counselling [8].

Beyond connection, social media can destigmatize mental health challenges through viral campaigns and influencer-driven content. Initiatives like #MentalHealthAwareness on Instagram have reached millions, encouraging adolescents to speak openly about anxiety or depression. In regions with cultural taboos around mental health, such as parts of South Asia or Sub-Saharan Africa, anonymous online spaces allow teens to seek advice without fear of judgment. Research from the UK’s Royal Society for Public Health underscores that moderate social media use can boost self-esteem through positive feedback and validation, provided interactions are supportive [9]. These benefits are not trivial; they address critical gaps in mental health infrastructure, especially in low-resource settings where trained professionals are scarce fewer than 2 psychiatrists per 100,000 people in many African nations, according to WHO data [10].

### Risks to adolescent mental health

Yet, the same platforms that uplift can also undermine adolescent well-being. Excessive social media use is linked to increased rates of anxiety, depression, and loneliness, with a 2019 meta-analysis showing a dose-response relationship teens spending over 3 h daily on social media were twice as likely to report poor mental health outcomes [11]. Cyberbullying is a pervasive threat; UNICEF reports that 1 in 3 young people globally have experienced online harassment, often leading to severe emotional distress or even self-harm [12]. Comparison culture, fuelled by curated images of perfection, distorts self-perception, particularly among girls, with studies linking Instagram use to body dissatisfaction and eating disorders [13].

Sleep disruption is another insidious risk. The blue light from screens and the addictive nature of scrolling driven by algorithms designed to maximize engagement interfere with adolescents circadian rhythms, a critical factor in emotional regulation during puberty. A 2021 study found that teens using social media within an hour of bedtime reported poorer sleep quality, correlating with higher anxiety levels [14]. Younger adolescents (12–15 years) are especially vulnerable, lacking the self-regulation to limit usage and being more susceptible to online peer pressure or trolling. In low-resource settings, where mental health support is often non-existent, these risks compound existing stressors like poverty or family instability, creating a vicious cycle. The evidence is clear: while social media offers benefits, its unchecked use poses threats to adolescent mental health, demanding attention.

### Disparities and contextual factors

The impact of social media on adolescent mental health is not universal; it varies across socioeconomic, cultural, and gender lines, often exacerbating inequities. In high-income countries, where over 90% of teens have personal devices, overexposure is a primary concern, with “digital addiction” linked to declining well-being [15]. Conversely, in low- and middle-income countries, “digital poverty” limits access UNICEF estimates that 2.2 billion children and youth lack reliable internet at home yet shared or intermittent use still exposes them to risks like cyberbullying without the protective buffer of digital literacy or parental oversight [16]. In Sierra Leone, for instance, teens in urban slums often access social media via cybercafés, encountering harmful content with little guidance, a contrast to wealthier peers with moderated home access. 

Cultural norms further shape social media’s impact. In some Middle Eastern or South Asian contexts, girls face stricter online monitoring due to gender norms, leading to stress from restricted expression, while boys may encounter pressure to engage in risky online behaviours to prove masculinity [17]. Gender-based cyberbullying also disproportionately targets girls, with a 2022 global survey finding that 60% of female teens reported receiving unwanted sexual messages online compared to 20% of boys [18]. These disparities underscore that social media’s mental health effects are not just about usage but about the broader social environment.

The post-pandemic context adds another layer of complexity. Lockdowns forced adolescents into digital spaces for schooling and socialization, blurring boundaries between necessary and excessive use. A 2022 WHO report noted a 25% rise in adolescent anxiety disorders globally post-COVID-19, partly attributed to prolonged screen time and social isolation transitioning into social media overuse [19]. “Zoom fatigue” from virtual learning often spilled into late-night scrolling as a coping mechanism, further disrupting mental health. In low-resource settings, where schools remain under-resourced post-pandemic, reliance on social media for information persists, amplifying exposure to misinformation or harmful trends without counterbalancing offline support.

These disparities underscore a critical point: social media’s impact on adolescent mental health cannot be addressed with a one-size-fits-all approach. Solutions must account for access gaps, cultural contexts, and the lingering effects of the pandemic, ensuring that interventions do not widen existing inequities but instead prioritize the most vulnerable teens.

### A multi-stakeholder framework for balance

Given the benefits and risks, a balanced approach to social media’s role in adolescent mental health requires coordinated action across sectors. I propose a multi-stakeholder framework with four interconnected pillars: parental and educator empowerment, tech industry accountability, clinical integration, and policy regulation. This framework aims to mitigate harm while preserving social media’s potential as a tool for well-being, tailored to diverse global contexts.

First, empowering parents and educators is foundational. Many adults lack the digital literacy to guide adolescents through online spaces safely. Programs should train them to set boundaries such as time limits or device-free zones and recognize warning signs like withdrawal or mood swings linked to social media stress. In Australia, the eSafety Commissioner’s resources for parents have shown promise, with 70% of participants reporting improved confidence in managing teens online activity [20]. Scaling such initiatives, even in low-resource settings through community centres or schools, can create a first line of defence.

Second, the tech industry must be held accountable. Social media platforms, driven by profit-oriented algorithms, often prioritize engagement over well-being. Companies like Meta and ByteDance should implement adolescent-specific safeguards, including default time limits, anti-bullying filters, and age-appropriate content moderation. Transparent reporting on mental health impacts akin to environmental impact assessments should be mandatory. The UK’s Online Safety Bill, which fines platforms for failing to protect minors, offers a model that could be adapted globally, pressuring tech giants to prioritize safety [21].

Third, clinical integration is critical. Paediatricians and mental health providers must screen for social media-related stressors during routine visits, using validated tools to assess usage patterns and emotional impact. Interventions, such as cognitive-behavioural strategies to combat online comparison or anxiety, should be tailored to adolescents needs. A 2022 study showed that integrating digital well-being into primary care increased early detection of mental health issues by 40% among teens [22]. Training clinicians, even via virtual modules in resource-limited areas, can bridge gaps in support.

Finally, policy and regulation must underpin these efforts. Governments should enact adolescent-focused digital policies, such as data privacy laws preventing exploitative targeting of teens and mandatory mental health impact assessments for social media apps. The European Union’s Digital Services Act, which holds platforms liable for harmful content, sets a precedent [23]. Globally, WHO and UNICEF could advocate for harmonized standards, while national policies adapt to local contexts ensuring, for instance, that rural teens in low-income settings are not excluded from protections due to access barriers.

Feasibility hinges on leveraging existing structures. Pilot programs in diverse settings urban centres in high-income countries, rural areas in low-income ones can test this framework, starting with community-driven digital literacy initiatives funded by public-private partnerships. GAVI Alliance’s model of scaling health interventions through phased rollouts offers a blueprint for managing costs and resistance [24]. The evidence is compelling: a 2020 review of multi-sectoral online safety programs found a 30% reduction in adolescent-reported online harm when stakeholders collaborated [25]. This framework is not a quick fix; it requires sustained commitment but promises a future where social media supports rather than undermines adolescent mental health.

## Limitations

This viewpoint draws on both global literature and local research at the Institute for Development in Sierra Leone. The local findings may not be generalizable to all adolescents in low- and middle-income countries. Additionally, the rapidly evolving nature of digital platforms means that trends described here may change over time. Future research with larger, more representative samples and longitudinal designs is recommended.

## Call to action and conclusion

The stakes of social media’s impact on adolescent mental health are staggering. Unchecked risks threaten to fuel a generational mental health crisis, with WHO projecting that 1 in 4 teens globally will experience a mental disorder by 2030 if trends persist [26]. Yet, ignoring the benefits risks cutting off vital support for vulnerable youth who rely on digital spaces for connection and resources. Post-pandemic, as digital habits solidify, we stand at a crossroads. This viewpoint’s multi-stakeholder framework offers a path to balance, but its success depends on urgent, collective action.

Governments must lead by funding digital literacy programs and enforcing protective regulations. National health and education ministries should integrate online safety into curricula and public health campaigns, ensuring no adolescent is left behind due to access or income. Tech companies bear a moral and practical responsibility to redesign platforms with adolescent well-being in mind, implementing safeguards over profit-driven algorithms starting with transparent impact assessments by 2026. Clinicians and educators, as frontline actors, must weave social media discussions into health checkups and classrooms, normalizing conversations about digital stress and equipping teens with coping tools. Researchers have a role too; longitudinal studies tracking social media’s evolving impact across cultures are essential to refine interventions.

The vision is clear: a digital world where social media empowers rather than harms, where a teen in rural Sierra Leone or urban London can log on without fear of bullying or burnout, finding instead a community that uplifts. In Sierra Leone, I have seen the transformative potential of digital access when guided safely resulting in teens using social media to access educational content rather than harmful trends. Scaling such grassroots efforts globally could turn the tide, ensuring that a teen in rural Sierra Leone not only logs on safely but thrives through digital empowerment. This is not just about technology it is about dignity, equity, and the right of every adolescent to thrive in an increasingly connected age. The post-pandemic surge in digital reliance is our wake-up call; inaction now risks embedding mental health inequities for decades.

Let us commit to small, deliberate steps piloting this framework in diverse settings with WHO and UNICEF backing, prioritizing the most vulnerable teens. The moral imperative is undeniable: we must transform the digital landscape into a safe space for the next generation’s mental health. The time for half-measures is over; together, we can build a future where every adolescents well-being is protected, online and off.

## Data Availability

No datasets were generated or analysed during the current study.

## References

[1] Pew Research Center. Teens, social media and technology 2021. Washington, DC: Pew Research Center; 2021.

[2] Blakemore SJ, Choudhury S. Development of the adolescent brain: implications for executive function and social cognition. J Child Psychol Psychiatry. 2006;47(3–4):296–312. 16492261 10.1111/j.1469-7610.2006.01611.x

[3] Santomauro DF, Herrera AM, Shadid J, Zheng P, Ashbaugh C, Pigott DM, Abbafati C, Adolph C, Amlag JO, Aravkin AY, Bang-Jensen BL. Global prevalence and burden of depressive and anxiety disorders in 204 countries and territories in 2020 due to the COVID-19 pandemic. Lancet. 2021;398(10312):1700–12. 34634250 10.1016/S0140-6736(21)02143-7 PMC8500697

[4] McGorry P, Gunasiri H, Mei C, Rice S, Gao CX. The youth mental health crisis: analysis and solutions. Front Psychiatry. 2025;15:1517533. 39906686 10.3389/fpsyt.2024.1517533 PMC11790661

[5] Twenge JM, Campbell WK. Associations between screen time and lower psychological well-being among children and adolescents: evidence from a population-based study. Prev Med Rep. 2018;12:271–83. 30406005 10.1016/j.pmedr.2018.10.003 PMC6214874

[6] Fegert JM, Gottschalk G, Schneider R, Sitarski E, Sounderajah V, Graham G. Navigating life transitions and mental wellbeing in the digital age: a call for stakeholders to embrace innovation and collaboration. Child Adolesc Psychiatry Mental Health. 2025;19(1):67. 10.1186/s13034-025-00932-2 PMC12166554 40517237

[7] GLSEN. Out online: the experiences of lesbian, gay, bisexual and transgender youth on the internet. New York: GLSEN; 2020.

[8] Anderson M, Jiang J. Teens’ social media habits and experiences. Washington, DC: Pew Research Center; 2020.

[9] Royal Society for Public Health. #StatusOfMind: social media and young people’s mental health and wellbeing. London: RSPH; 2017.

[10] World Health Organization. Mental health atlas 2020. Geneva: WHO; 2020.

[11] Marino C, Gini G, Vieno A, Spada MM. The associations between problematic Facebook use, psychological distress and well-being among adolescents and young adults: a systematic review and meta-analysis. J Affect Disord. 2018;226:274–81. 29024900 10.1016/j.jad.2017.10.007

[12] UNICEF. Cyberbullying: what is it and how to stop it. New York: UNICEF; 2021.

[13] Fardouly J, Vartanian LR. Social media and body image concerns: current research and future directions. Curr Opin Psychol. 2016;9:1–5.

[14] Scott H, Biello SM, Woods HC. Social media use and adolescent sleep patterns: cross-sectional findings from the UK millennium cohort study. BMJ Open. 2021;11(9):e049826. 10.1136/bmjopen-2019-031161 PMC6830469 31641035

[15] Vogels EA, Gelles-Watnick R. Teens and social media: Key findings from Pew Research Center surveys. Washington, DC: Pew Research Center; 2023.

[16] UNICEF. Digital connectivity during COVID-19: 2.2 billion children and young people lack internet access at home. New York: UNICEF; 2021.

[17] Livingstone S, Stoilova M. Global kids online: comparative report. London: London School of Economics; 2019.

[18] Plan International. Free to be online? Girls’ and young women’s experiences of online harassment. Woking: Plan International; 2022.

[19] World Health Organization. Mental health of adolescents post-COVID-19: global burden and trends. Geneva: WHO; 2022.

[20] eSafety Commissioner. Parental guidance: helping young people stay safe online. Canberra: Australian Government; 2021.

[21] UK Government. Online safety bill: policy overview. London: Department for Digital, Culture, Media & Sport; 2022.

[22] Stewart E, Milton A, Yee HF, Song MJ, Roberts A, Davenport T, Hickie I. eHealth tools that assess and track health and well-being in children and young people: systematic review. J Med Internet Res. 2022;24(5):e26015. 35550285 10.2196/26015 PMC9136648

[23] European Commission. Digital services act: ensuring a safe and accountable online environment. Brussels: European Commission; 2022.

[24] GAVI Alliance. GAVI strategy 2021–2025: leaving no one behind with immunization. Geneva: GAVI; 2020.

[25] Stoilova M, Nandagiri R, Livingstone S. Children’s Understanding of personal data and privacy online: a systematic review. Inf Commun Soc. 2020;23(14):2001–18.

[26] World Health Organization. Adolescent mental health: global action plan 2013–2030. Geneva: WHO; 2021.

